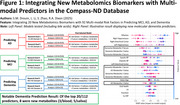# Integrating Novel Metabolomics Biomarkers with Multi‐modal Risk Factors in Predicting Mild Cognitive Impairment and Alzheimer's Disease

**DOI:** 10.1002/alz70856_104500

**Published:** 2025-12-26

**Authors:** Roger A. Dixon, Shannon M. Drouin, Linzy Bohn, Shuang Zhao, Liang Li

**Affiliations:** ^1^ University of Alberta, Edmonton, AB, Canada

## Abstract

**Background:**

Recent research approaches to an expanding collection of well‐characterized databases have examined varying arrays of biomarkers and risk factors related to Alzheimer's disease (AD). Analytical techniques that simultaneously evaluate relative importance of numerous candidate factors can successfully identify leading predictors within multiple‐variable, high‐dimensional datasets. Our objective was to integrate new molecular biomarkers with established AD‐related biomarkers and risk factors to evaluate their relative predictive contributions using a series of machine learning classifier models.

**Method:**

We assembled 92 AD‐related indicators representing 10 key modalities of AD risk (demographic, biomarkers, lifestyle, sensory, vascular, imaging, functional, psychiatric, anthropometric, clinical health) for participants from three cohorts in the Canadian Consortium on Neurodegeneration in Aging database (aka COMPASS‐ND): (1) Cognitively Unimpaired (CU), (2) Mild Cognitive Impairment (MCI), (3) AD. Each cohort was divided for separate 3‐group training and validation analyses. Novelty: High‐throughput metabolomics analyses were conducted on both serum and saliva, each analysis detecting over 7000 metabolite peaks. Leading molecular predictors for the two‐group comparisons and two AD‐related bile acids produced 20 novel predictors. Data‐driven analyses (in Python 3.9) included three machine learning classifier algorithms (eg, sklearn RandomForestClassifier) processing 112 (92+20) predictors.

**Result:**

Three sets of analyses (same predictors) were conducted (Predicting AD (CU‐AD); Predicting MCI (CU‐MCI); Predicting dementia (MCI‐AD)). All models performed successfully in the main metrics (eg, Accuracy, Precision; AUC range: 0.81 – 0.98). Key results displayed in Explainable AI Tree SHAP plots vividly depicted discriminating patterns of leading multi‐modal predictors. Commonly observed predictors were from new metabolomics analyses (saliva, blood), imaging, vascular, AD biomarkers, sensory, clinical health and lifestyle. Figure 1 displays the overall models tested (left panel) and the results for Predicting Dementia (right panel). A striking result is that of 112 predictors, the top 20 included 8 newly discovered metabolites, 5 of which were from salivary analyses.

**Conclusion:**

New molecular biomarkers (from both blood and saliva) perform strongly when integrated into databases with established AD biomarkers and risk factors. Implications include (1) enhancing identification of early mechanistic pathways, (2) importance of conducting integrative and interactive biomarker analyses, especially data‐driven, and (3) potential for non‐invasive salivary biomarkers for promoting assessment accessibility across diverse communities.